# Reference range of liver corrected T1 values in a population at low risk for fatty liver disease—a UK Biobank sub-study, with an appendix of interesting cases

**DOI:** 10.1007/s00261-018-1701-2

**Published:** 2018-07-21

**Authors:** A. Mojtahed, C. J. Kelly, A. H. Herlihy, S. Kin, H. R. Wilman, A. McKay, M. Kelly, M. Milanesi, S. Neubauer, E. L. Thomas, J. D. Bell, R. Banerjee, M. Harisinghani

**Affiliations:** 1 Division of Abdominal Imaging, Massachusetts General Hospital, Boston, MA USA; 2 Perspectum Diagnostics, Oxford, UK; 3 Oxford Centre for Clinical Magnetic Resonance Research, University of Oxford, Oxford, UK; 4 Department of Life Sciences, University of Westminster, London, UK

**Keywords:** Non-alcoholic fatty liver disease, Multiparametric magnetic resonance imaging, Corrected T1, Quantitative imaging biomarkers

## Abstract

**Purpose:**

Corrected T1 (cT1) value is a novel MRI-based quantitative metric for assessing a composite of liver inflammation and fibrosis. It has been shown to distinguish between non-alcoholic fatty liver disease (NAFL) and non-alcoholic steatohepatitis. However, these studies were conducted in patients at high risk for liver disease. This study establishes the normal reference range of cT1 values for a large UK population, and assesses interactions of age and gender.

**Methods:**

MR data were acquired on a 1.5 T system as part of the UK Biobank Imaging Enhancement study. Measures for Proton Density Fat Fraction and cT1 were calculated from the MRI data using a multiparametric MRI software application. Data that did not meet quality criteria were excluded from further analysis. Inter and intra-reader variability was estimated in a set of data. A cohort at low risk for NAFL was identified by excluding individuals with BMI ≥ 25 kg/m^2^ and PDFF ≥ 5%. Of the 2816 participants with data of suitable quality, 1037 (37%) were classified as at low risk.

**Results:**

The cT1 values in the low-risk population ranged from 573 to 852 ms with a median of 666 ms and interquartile range from 643 to 694 ms. Iron correction of T1 was necessary in 36.5% of this reference population. Age and gender had minimal effect on cT1 values.

**Conclusion:**

The majority of cT1 values are tightly clustered in a population at low risk for NAFL, suggesting it has the potential to serve as a new quantitative imaging biomarker for studies of liver health and disease.

Liver disease is rapidly becoming one of the major causes of mortality in the Western world, fueled by the growing pandemic of obesity. There is increasing awareness that excessive liver fat (termed steatosis, or non-alcoholic fatty liver—NAFL) is also a precursor to liver inflammation (non-alcoholic steatohepatitis—NASH), fibrosis, and cirrhosis, which are also associated with cardiovascular disease, further contributing to the overall mortality [1–3]. Moreover, NASH and liver fibrosis predispose individuals to hepatocellular carcinoma [4, 5]. Williams et al. showed that the United States prevalence rates of NAFL and NASH were 46% and 12%, respectively [6]. Unfortunately, liver disease is frequently not detected until clinical symptoms appear, when fewer treatment options exist, and many of those with liver disease are unaware of the fact [7]. It is therefore essential to identify those individuals at highest risk of progressing to cirrhosis while the disease is in its early stages.

Multiparametric magnetic resonance imaging (MRI) of the liver has been shown to have prognostic capabilities in the setting of chronic liver disease, as well as being able to detect early stages of liver fibrosis [8]. This technique has been validated against biopsy and has an advantage over conventional MRI or ultrasound in that it is quantitative, referring to absolute rather than relative values. Moreover, parametric maps of proton density fat fraction or T1 relaxation times (a measure of free water content) can be compared objectively across populations and longitudinally within individuals. This multiparametric MRI data can be processed to provide key quantitative liver metrics, including liver fat, iron content, and the novel Liver Inflammation and Fibrosis (LIF) score, which is based on iron corrected T1 (cT1). cT1 is a novel imaging biomarker based on T1 mapping technology, which takes advantage of the increases in extracellular tissue fluid that occur in response to inflammation and fibrosis, while also correcting for elevated iron content. In a recent clinical trial using a pre-standardized version of the technology, high cT1 values (> 875 ms) were shown to strongly predict liver-related clinical outcomes (e.g., hepatic encephalopathy, variceal bleeding, ascites, death) [9]. Although not definitively established, the generally accepted upper limit of normal for liver fat content, as measured by proton density fat fraction (PDFF), is approximately 5% [10–12]. Similar population-based ranges and cut-off data for the cT1 biomarker are needed. There is a need for an understanding of normality and abnormality in the context of NAFL and NASH, to establish which populations are at risk, and, future criteria for treatment with a variety of NASH agents, which are currently being developed by the pharmaceutical industry.

The purpose of this article is to provide a set of reference values for cT1 within a population nominally at low risk for fatty liver disease. To this end, we use data acquired as part of the largest prospective population study in history, UK Biobank [13–15]. In this study, we identify the subset of the UK Biobank cohort at ‘low risk’ for NAFL and NASH and describe the range of cT1 values in this population. We also define the effects of age and gender on cT1 values.

## Materials and methods

### Study design and population

**Fig. 1:**
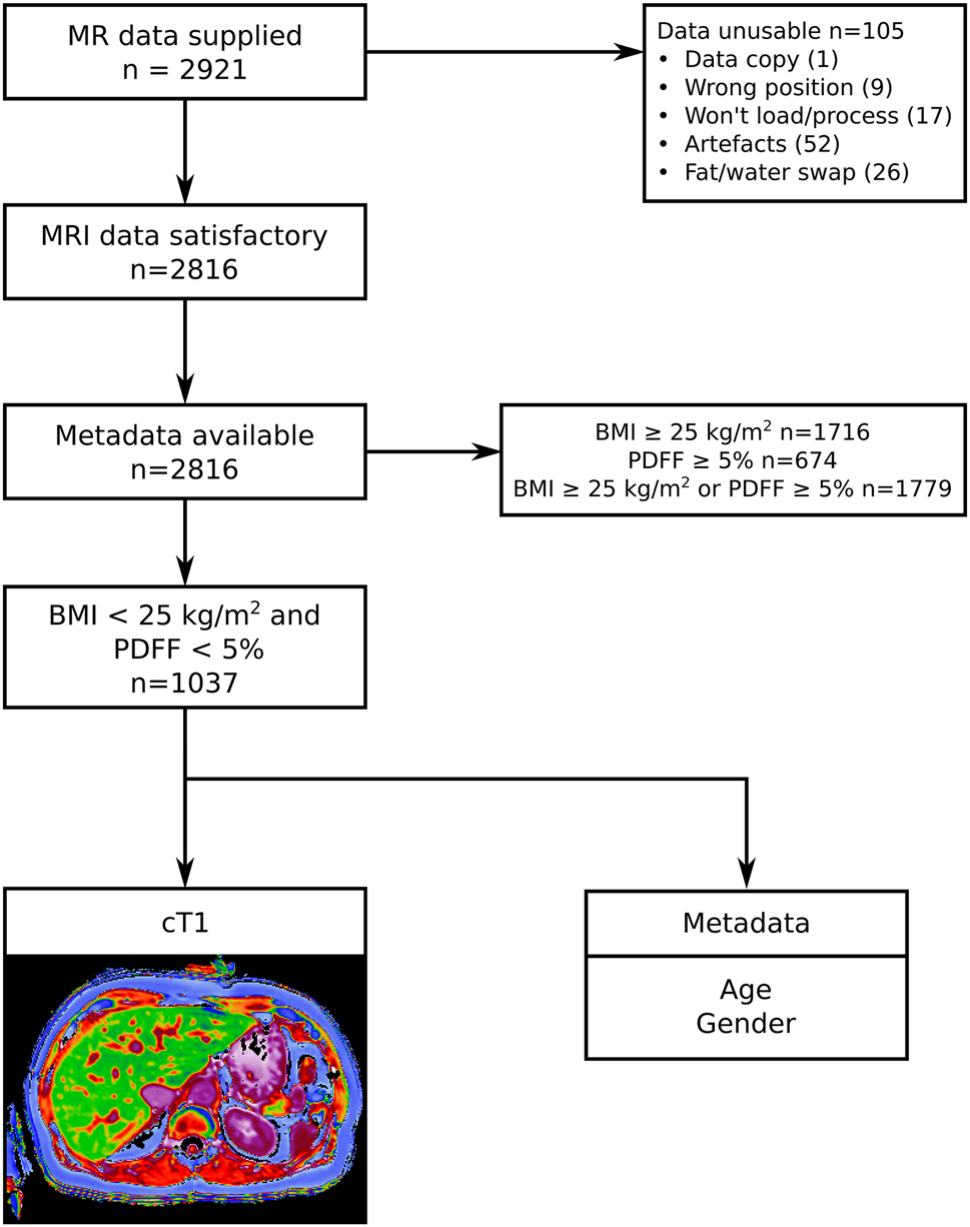
Study design

This was a retrospective study of MR data acquired as part of the UK Biobank imaging enhancement study between August 2014 and August 2015. The UK Biobank study recruited 500,000 volunteers between the ages of 40 and 69 years from across the UK between April 2007 and August 2010, based on proximity to the 21 assessment centers and registration with the National Health Service [16]. A random subset of 100,000 subjects are being invited to participate in the imaging sub-study that images the brain, heart, abdomen, bones, and carotid arteries of these subjects [17]. At the time this retrospective study was performed, scan data for 2921 subjects were available for processing. 6000 subjects had been scanned in the pilot phase, but following review of the pilot data, the MR sequence was further optimized. The 2921 subjects in this study were scanned between January 4, 2016 and September 8, 2016. The study protocol is shown in Fig. 1. Of the 2921 datasets available for processing, 105 (~ 4%) were deemed incomplete or of insufficient quality. As outlined in Fig. 1, the most common reason for failure was generally related to motion artifacts. This left 2816 (~ 96%) datasets that were analyzed to obtain quantities for liver fat fraction (proton density fat fraction—PDFF) and fibroinflammatory disease (cT1). Accompanying participant data [gender, age, and body mass index (BMI)] were acquired through a data access application to UK Biobank (Application 9914). The reference population was defined by excluding those participants with either PDFF ≥ 5%, or BMI ≥ 25 kg/m^2^. Data were analyzed for the reference cohort, comparison cohorts with PDFF ≥ 5% and/or BMI ≥ 25 kg/m^2^, as well as separately for gender and age ranges of 40–49, 50–59, 60–69, and ≥ 70 years.

### Imaging protocol

Image acquisition took place at the dedicated Biobank imaging Centre at Cheadle (UK) using a Siemens 1.5 T MAGNETOM Aera, syngo MR D13. Two sequences were used to acquire data: a Shortened Modified Look Locker Inversion (ShMOLLI) [18] and a multiecho spoiled-gradient-echo [19].

A single transverse slice, located at the porta hepatis, was chosen to represent the liver for both acquisitions. Acquisition was performed in end-expiration breath-hold and without the aid of any contrast agent injection. This slice-based methodology has previously been shown to correlate well with histology [8] and predict liver-related outcomes [9]. The rapid acquisition time (≤ 3 min) was an essential requirement to meet the high-throughput demands of the UK Biobank study [14].

The ShMOLLI sequence is based on an ECG-Triggered method that samples the T1 recovery curve in three epochs over 9 heartbeats using single-shot balanced steady-state free precession (bSSFP) acquisitions with the following parameters: TR = 4.8 ms, TE = 1.93 ms, flip angle = 35°, slice thickness = 8 mm, field of view = 44 × 33 cm^2^, acquisition matrix = 192 × 144 (zero padded to 384 × 288) yielding an interpolated pixel size of 1.1 mm × 1.1 mm. Parallel imaging acceleration factor of 2 with 24 reference lines was used with the integrated Parallel Acquisition Technique (iPAT). Trigger delay was set to 50 ms and the first ShMOLLI inversion time (TI) was set to 170 ms with five increments of 50 ms each.

The T1 relaxation map acquired using the ShMOLLI sequence is affected by excess iron, which reduces T1. Thus, an algorithm removes the bias introduced by excess iron, which can be calculated from the T2* maps [20], from the T1 measurements, providing the iron corrected T1 (cT1) [21]. The specific methodology of this algorithm has been described previously [8], and simulates the T1 that would be measured on a 3T scanner in the absence of excess liver iron, with a T2* of 23.1 ms (at 1.5T) used as the threshold for excess iron.

The multiecho spoiled-gradient-echo chemical shift encoded acquisition was used to calculate T2* and PDFF maps of the liver. The following parameters were used: field of view = 40 × 40 cm^2^, acquisition matrix = 160 × 160 yielding a voxel size of 2.5 mm  ×  2.5 mm, slice thickness = 6 mm, flip angle = 20°, TR = 27 ms, and 2 signal averages, requiring 9 s to acquire. Ten echo times were selected such that the signals from fat and water were in phase and out of phase at 1.5T (TE = 2.38, 4.76, 7.14, 9.52, 11.90, 14.28, 16.66, 19.04, 21.42, and 23.80 ms). For PDFF, a three-point DIXON technique [22, 23] was used on the complex data from the second, third, and fourth echoes. This technique takes into account magnetic field inhomogeneity and assumes that the fat has a single-peak frequency. The flip angle of 20 degrees leads to some T1 bias, which has been shown to reduce PDFF values by a factor of 1.2, relative to using a lower flip angle with an in-house implementation of the IDEAL methodology [24]. For R2* estimation, an exponential signal decay model [25] was fitted to the magnitude data from the second, fourth, sixth, eight, and tenth echo times (i.e., the five in-phase echoes).

### Post-processing

Raw MR data were sent to a central reporting laboratory and transferred to an individual workstation (Mac OSX) for analysis. All processed data are available through application to UK Biobank.

### Image analysis

Image data were analyzed, blinded to all other subject data, using Liver*MultiScan* Discover software [22]. For each T2*, cT1, and PDFF image, three circular regions of interest (ROIs) of 15 mm diameter were selected, and a mean value from the pixels within the ROI was calculated. ROIs were manually placed by a trained analyst to capture the distribution of image intensities within the liver and avoid MRI artifacts, if present. The T2* and T1 values together provide the cT1. Characterization of liver fat (PDFF) in the UK Biobank cohort has been previously reported [26].

### Inter- and intra-reader variability

Inter- and intra-reader variability in ROI placement was determined in an initial set of MR datasets collected from the UK Biobank imaging cohort. 39 MR datasets were selected to encompass the range of cT1 and PDFF values found in the population. Six technologists analyzed the anonymized datasets on day one and then again on day four. Intra and inter-reader agreement was calculated using intra-class correlation (two-way mixed model with fixed effects, referred to as Class 3 in the original work) [27] and Bland–Altman analyses [28].

Two sets of Bland–Altman analyses were performed. The first set compared values acquired on day one and day four for each of the six technologists. The mean and range of the limits of agreement from these six Bland–Altman are reported to demonstrate the intra-rater reliability. The second set of Bland–Altman analyses was an all-against-all analysis of data from day one. The results of each technologist were compared to those of each other technologist. The mean and range of the limits of agreement from these fifteen Bland–Altman analyses are reported to demonstrate the inter-rater reliability.

### Statistical analysis

Normality of BMI, PDFF, and cT1 distributions was tested using D’Agostino–Pearson and Kruskal–Wallis tests. Summary data for cT1 are presented as medians with interquartile ranges (IQR) and means with standard deviation (SD). Mann–Whitney tests were used to test for statistically significant differences between groups. Correlation analysis was performed using linear regression.

### Case studies

Case studies are provided in the Appendix. Case studies given in Figs. 4, 5, 7, and 8 in Appendix are from the Patient Understanding of Liver*MultiScan* trial (ClinicalTrials.gov Identifier: NCT02877602) performed at the Oxford University NHS Foundation Trust, UK. The case study in Fig. 6 is from Massachusetts General Hospital, US.

## Results

### cT1 distribution in low-risk subpopulation

**Fig. 2:**
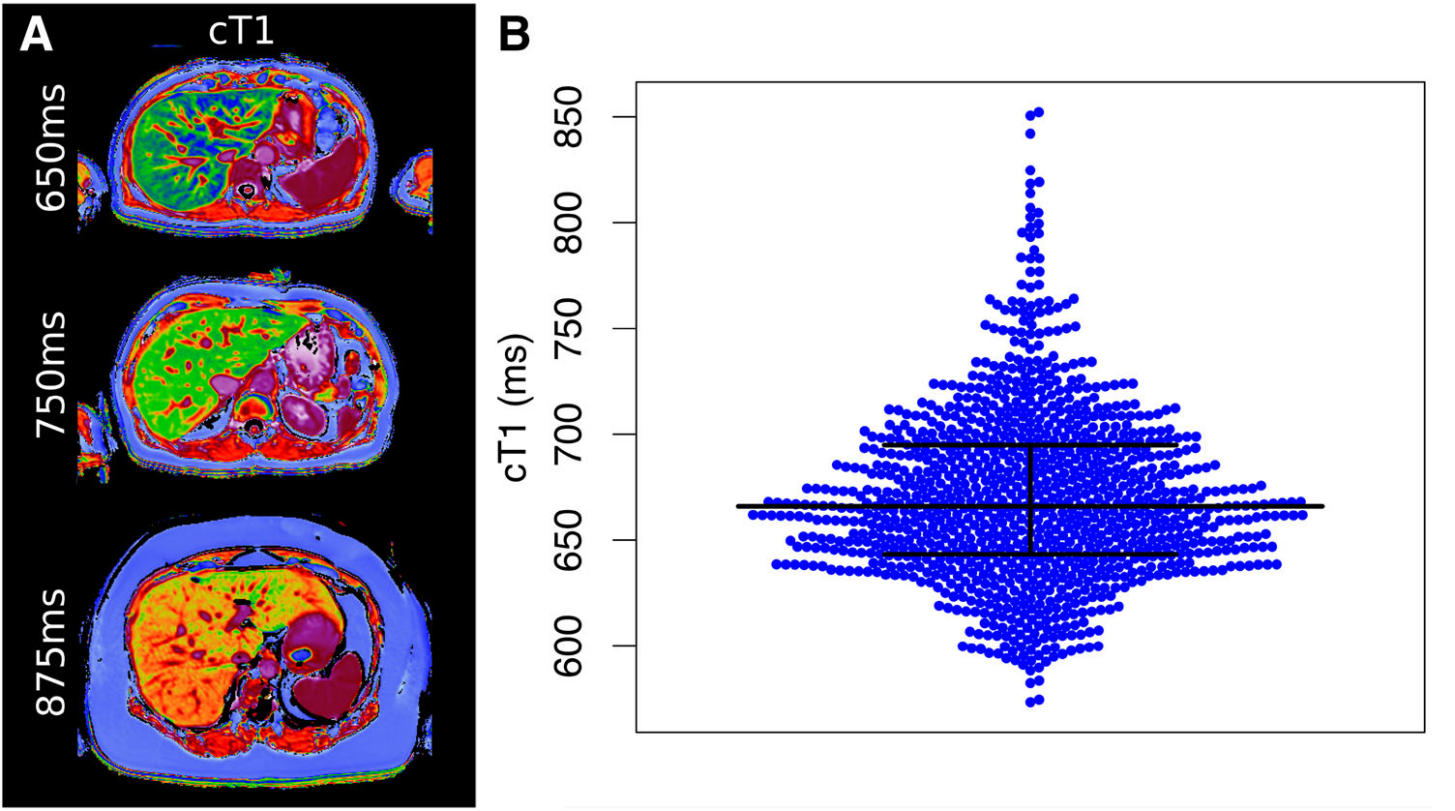
Range of cT1 in the reference population. **A** Example images with three different cT1 values. **B** Beeswarm plot showing distribution of cT1 values in the reference population, with median (666.0 ms) and IQR (643.3–694.8 ms) shown

Figure 2A shows example cT1 images. Figure 2B shows the range of cT1 values in the reference population. cT1 values are non-normally distributed with a median value of 666.0 ms (IQR 643.3–694.8 ms; 2.5–97.5 percentiles of 600–763 ms) (Fig. 2). Subcohorts were defined as non-overweight (BMI < 25 kg/m^2^, 39% of the total cohort), non-steatotic (PDFF < 5%, 76% of the total cohort), and ‘reference’ (both non-overweight (BMI < 25 kg/m^2^) and free from steatosis (PDFF < 5%), 37% of the total cohort). Summary statistics for cT1 in these three cohorts are shown in Table 1. For comparison, Table 1 also describes the same statistics for three additional cohorts: non-overweight but steatotic (median cT1 of 712.5 ms), overweight but non-steatotic (median cT1 of 681.7 ms), and overweight and steatotic (median cT1 of 744.7). In each of these higher-risk cohorts, the median cT1 is significantly higher than the reference cohort (*p* < 0.0001). T2* values for 36.5% of the reference population (379/1037) were sufficiently low (< 23.1 ms; corresponding to elevated iron concentration) to require iron correction of T1 (cT1). For comparison, 42.2% of the original population (1187/2816) required iron correction of T1. It should be noted that this threshold for iron correction is lower than that which would be considered clinically significant iron overload.

**Table 1:** Summary statistics for cT1 (ms) in cohorts defined by BMI and PDFF

	*n*	% of total cohort (2816)	Min	25th Percentile	Median	75th Percentile	Max
BMI < 25 kg/m^2^ and PDFF < 5%	1037	37	573.4	643.3	666.0	694.8	852.2
BMI < 25 kg/m^2^	1100	39	573.4	644.7	667.6	697.9	882.7
PDFF < 5%	2142	76	554.5	649.3	674.0	703.6	852.2
BMI < 25 kg/m^2^ and PDFF ≥ 5%	63	2	648.4	684.5	712.5	748.2	882.7
BMI ≥ 25 kg/m^2^ and PDFF < 5%	1104	39	554.5	657.5	681.7	711.0	844.2
BMI ≥ 25 kg/m^2^ and PDFF ≥ 5%	611	22	618.8	711.0	744.7	792.4	990.5

The reference population is defined as BMI <25 kg/m^2^ and PDFF < 5%

In the original population, cT1 showed a significant but weak correlation with PDFF (*r*^2^ = 0.45; *p* < 0.0001) and a significant but very weak correlation with T2* (*r*^2^ = 0.004; *p* < 0.001). PDFF showed a significant but very weak correlation with T2* (*r*^2^ = 0.05; *p* < 0.0001).

### Gender and age characteristics of reference subpopulation

**Fig. 3:**
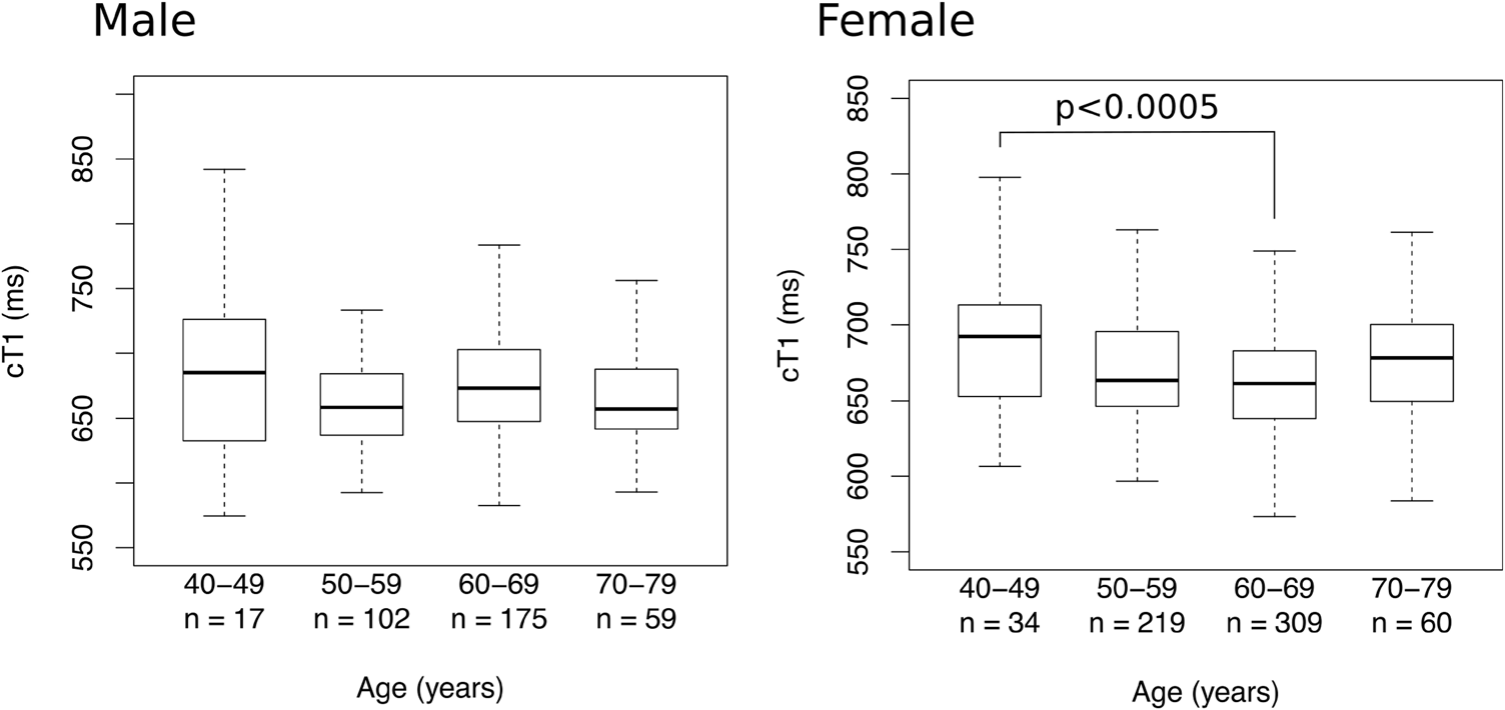
Boxplots showing distribution of cT1 in the reference population by age and gender. 62 individuals are not plotted as their age was missing from the dataset. Outliers not shown

**Fig. 4:**
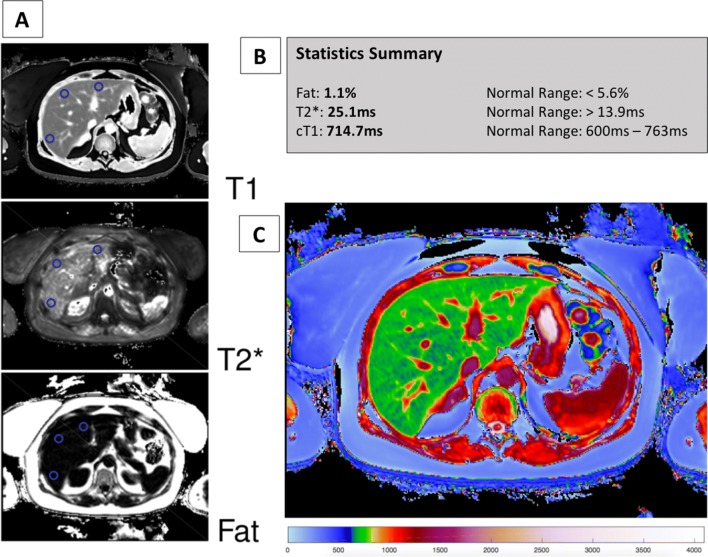
Example case provided for an overview of the software results sheet. The results sheet depicts source images (**A**) with circular regions of interest (ROI) drawn (blue circles). The statistics summary (**B**) provides values for calculated proton density fat fraction (PDFF), T2*, and cT1 (with normal reference ranges for each). The cT1 value is calculated from a circular ROI which should be placed within hepatic parenchyma, avoiding any vessels. The results sheet also includes a colormap (**C**) for a pictorial representation of cT1

**Fig. 5:**
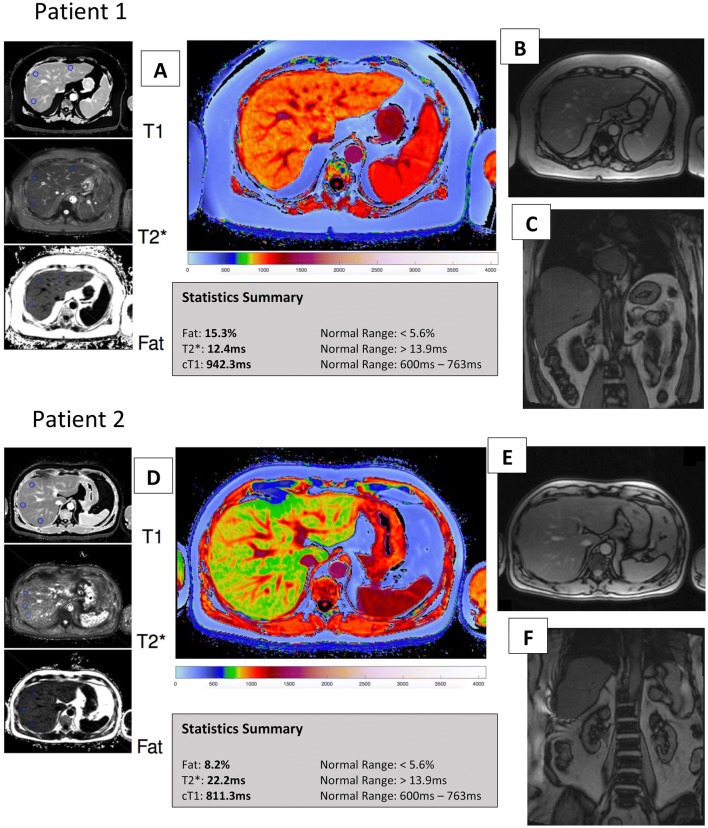
Cases 1 and 2: Patient 1 is a 64-year-old female with non-alcoholic steatohepatitis and patient 2 is a 42-year-old male with steatosis. Axial (**B**, **E**) and coronal (**C**, **F**) T2* weighted images of the liver provide anatomical information for each patient, but no sensitivity to detect underlying parenchymal disease. Results sheets for patient 1 (**A**) and 2 (**D**) demonstrate that both patients have elevated PDFF values, 15.3% for patient 1 and 8.2% for patient 2. An advantage of this method is that the level of fat, as well as its burden on the hepatic parenchyma, is instantly assessed. The colormaps demonstrate that the cT1 value of patient 1 is notably higher than patient 2, indicating a greater degree of inflammation and fibrosis. Patient 2 has a mildly elevated cT1 value, but has the opportunity for intervention (medical and/or lifestyle) before potentially progressing to the cT1 value of patient 1

**Fig. 6:**
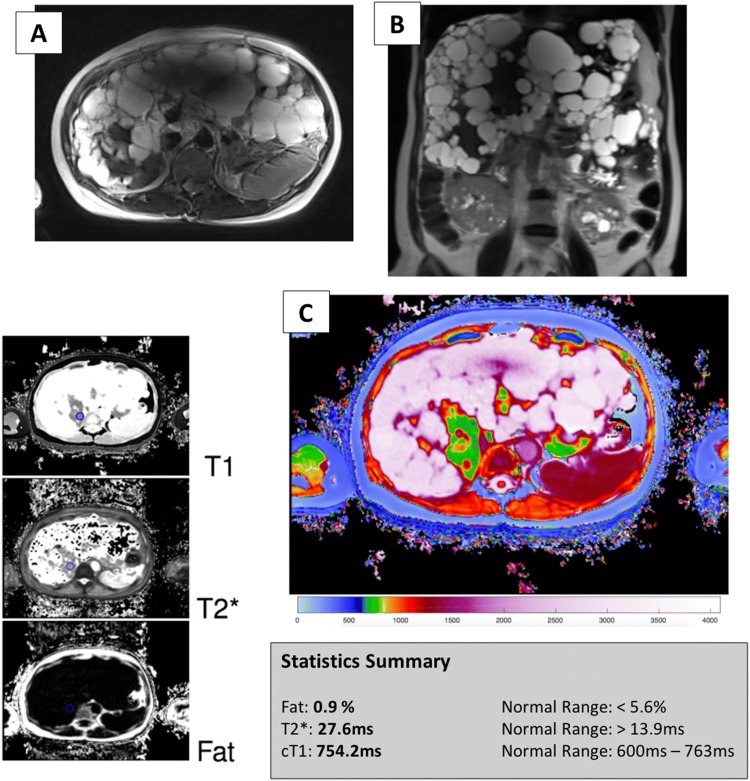
Case 3: A 47-year-old female with polycystic liver disease listed for liver transplant. MRI was obtained to assess hepatic cyst burden and integrity of the remaining hepatic parenchyma. Anatomical axial (**A**) and coronal (**B**) T2-weighted images of the abdomen demonstrate innumerable cysts throughout the liver. Results sheet (**C**) demonstrates that liver fat, T2*, and cT1 are all within normal limits. The results are also displayed in pictorial format with the areas of remaining hepatic parenchyma coded in green (normal cT1) on the colormap. This case uniquely exemplifies the advantage of this methodology over other techniques to assess the pathophysiological state of hepatic parenchyma, such as ultrasound or MR elastography. These elastography methods would produce spurious results in this setting, as the extensive cyst burden would alter the acoustic wave propagation through the liver

**Fig. 7:**
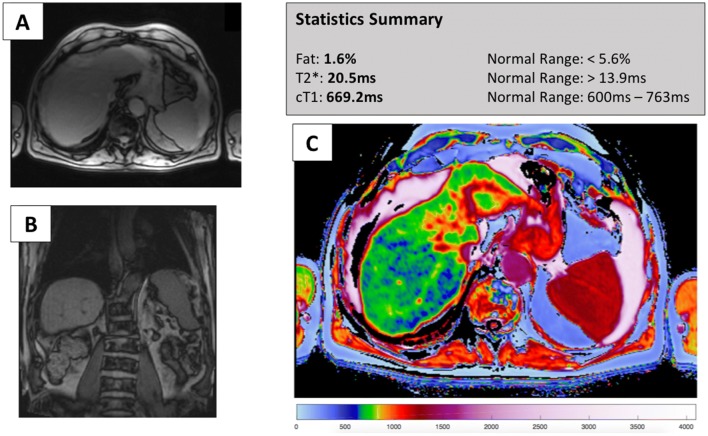
Case 4: A 79-year-old male with autoimmune hepatitis (AIH). Axial (**A**) and coronal (**B**) images of the liver provide anatomical information, but are unable to provide any clarity regarding the underlying pathophysiologic process. Results sheet (**C**) shows the complexity of this individual’s disease. In a pattern characteristic of AIH, the spread of fibroinflammatory disease is heterogeneous with some areas clearly demonstrating higher cT1 values than others. Measuring cT1 in a single circular region of interest would yield varying results (e.g., normal in the right lobe as shown here, and abnormal in the left lobe). The colormap image also clearly demonstrates the size of the complicating ascites (purple/pink), a source of discomfort for the patient. Consequently, the results sheet and images provide a comprehensive assessment of this patient’s complex disease

**Fig. 8:**
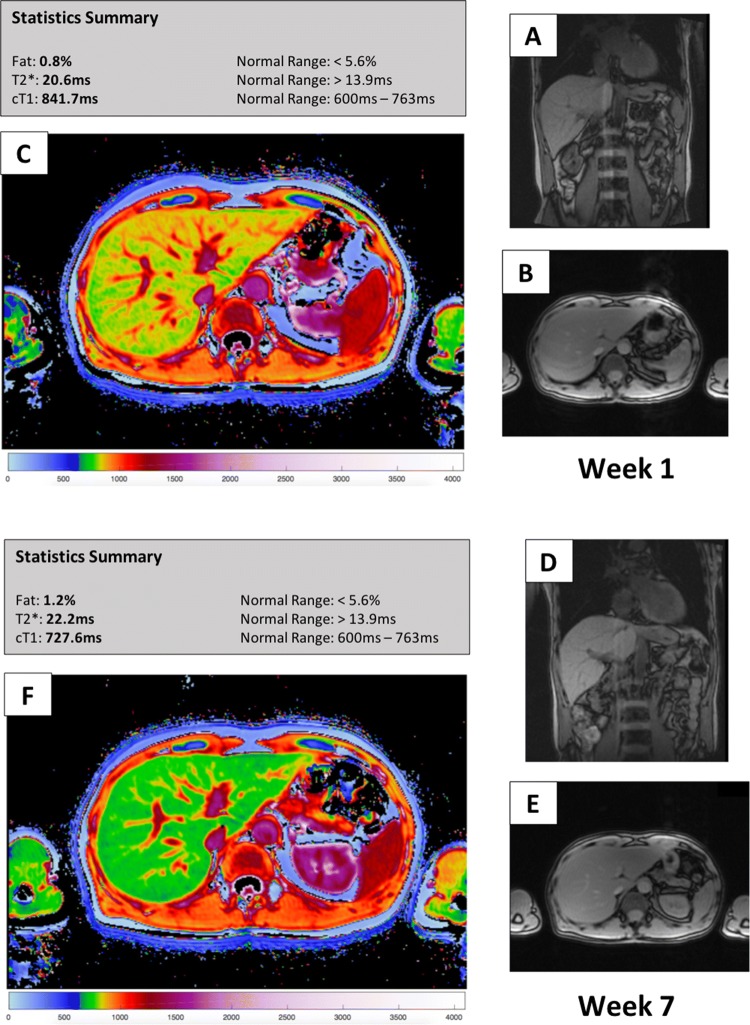
Case 5: A 51-year-old male who had lived with hepatitis C virus infection since 1984. Patient had failed to get a sustained virologic response (SVR) after interferon treatment in 2014, and is now undergoing treatment with ledipasvir/sofosbuvir. Axial (**B**, **E**) and coronal (**C**, **F**) T2*-weighted images of the liver provide anatomical information but are unable to provide further tissue characterization. Result sheets from examinations at the 1st (**A**) and 7th (**B**) weeks of treatment are provided. These clearly show response to the treatment, with the cT1 value and cT1 colormap improving over the course of treatment. Patient achieved SVR at 12 and 24 weeks

Table 2 and Fig. 3 show the breakdown of cT1 values by age and gender in the reference cohort. Overall, the effect size of age and gender was minimal. The cT1 values were lower in women than men, but not significantly so (median 664.5 vs. 671.7 ms, *p* = 0.11, Mann–Whitney test). Median cT1 values in different age groups showed minimal variation (from 662.4 to 691.9 ms).

**Table 2:** Median cT1 of reference cohort by age and gender

Group	Number	Median cT1 (IQR) by age group				
		40–49 years	50–59 years	60–69 years	70–79 years	All ages
All	1037	691.9 (650.5–721.9)	662.4 (644.1–692.5)	665.8 (640.2–691.5)	667.7 (644.6–695.9)	666.0 (643.3–694.8)
Male	384	685.3 (632.6–726)	658.5 (637.3–684.4)	673.2 (647.6–702.7)	657.1 (641.8–687.9)	671.7 (643.6–698.1)
Female	653	692.4 (654.5–711.8)	663.6 (646.5–695.6)	661.5 (638.2–682.8)	678.2 (650.3–699.8)	664.5 (643.3–692.2)

Among men, there was no difference between age groups. However, among women, there was a significant, though numerically small (median 692.4 vs. 665.8 ms), difference between the ages of 40–49 years and 60–69 years (*p* < 0.0005) (Fig. 3). Among women, there was a negative correlation between age and cT1 (*r* = − 0.12, slope (95% CI) − 0.67 ms/year (− 1.12 to − 0.23), *p* = 0.0029). No such correlation was evident among men. Compared to the whole Biobank population, this low-risk cohort is older and has a higher proportion of women (Table 3).

**Table 3:** Gender and age profiles of total and reference populations

	% of cohort	Ratio (female:male)	Mean age	Standard deviation
All (*n* = 2816)				
Male	48	1.1:1	62.1	7.1
Female	52		61.2	6.8
Reference (*n* = 1037)				
Male	37	1.70:1	62.0	7.1
Female	63		61.0	6.8

### Inter- and intra-reader variation in cT1 and PDFF

Intra-reader reproducibility for cT1 and PDFF readings is summarized in Table 4. Intra-class correlation was very high for both cT1 and PDFF. Intra-rater limits of agreement were in the range of ± 25 ms (cT1) and ± 0.5% (PDFF) for the six technologists. Inter-rater limits of agreement spanned approximately 60 ms (i.e., upper and lower limits of ± 30 ms) and 1.2% (i.e., upper and lower limits of ± 0.6%) for cT1 and PDFF ,respectively, and the magnitude of the largest bias between any pair of technologists was 15 ms for cT1 and 0.08% for PDFF.

**Table 4:** Reproducibility of cT1 and PDFF observations

	Intra-class correlation [µ (95%CI)]	Intra-rater lower limit of agreement [mean (range)]	Intra-rater upper limit of agreement [mean (range)]	Inter-rater lower limit of agreement [mean (range)]	Inter-rater bias [mean (range)]	Inter-rater upper limit of agreement [mean (range)]
PDFF	0.97 (0.96–0.99)	− 0.48% (− 0.62 to − 0.44%)	0.52% (0.37–0.61%)	− 0.57% (− 0.77 to − 0.40%)	0.01% (− 0.08 to 0.08%)	0.59% (0.35–0.78%)
cT1	0.93 (0.89–0.97)	− 25 ms (− 30 to − 17 ms)	20 ms (17–25 ms)	− 32 ms (− 52 to − 18 ms)	0 ms (− 13 to 15 ms)	31 ms (20–55 ms)

Intra-rater limits of agreement are a summary of six Bland–Altman analyses. Inter-rater limits of agreement are a summary of fifteen Bland–Altman analyses

For individual readers reading cT1 data on different days, the intra-class correlation was 0.93 (95% confidence interval 0.89–0.96). For individual readers reading the cT1 data on different days, the mean upper and lower limits of agreement from the six intra-rater analyses were 20 ms (range 17–25 ms) and − 25 ms (range − 30 to − 17 ms). For the fifteen inter-rater analyses, the mean upper and lower limits of agreement were 31 ms (range 20–55 ms) and − 32 ms (range − 52 to − 18 ms), and the mean bias was 0 ms (range − 13 to 15 ms).

For individual readers reading PDFF data on different days, the intra-class correlation was 0.97 (95% CI 0.96–0.99). For individual readers reading the PDFF data on different days, the mean upper and lower limits of agreement for the six intra-rater analyses were 0.52% (range 0.37–0.61%) and − 0.48% (range − 0.62 to − 0.44%). For the fifteen inter-rater analyses, the mean upper and lower limits of agreement were 0.59% (range 0.35–0.78%) and − 0.57% (range − 0.77 to − 0.40%), and the mean bias was 0.01% (range − 0.08 to 0.08%).

## Discussion

Liver disease is a growing clinical problem being fuelled by the obesity epidemic. Obesity is a risk factor for fatty liver disease, which has been defined as hepatic fat content greater than 5% [12]. Importantly, liver disease, unlike chronic pulmonary, cardiac, or neurodegenerative diseases, is usually asymptomatic, with few physical manifestations until the development of cirrhosis; thus, detection and case-finding cannot be based on clinical history and examination alone. Scaglione et al. estimated the prevalence of cirrhosis in the United States to be approximately 0.27% from NHANES and death registry data 1999–2010, corresponding to 633,323 adults. Mortality was 26.4% per 2-year interval in cirrhosis compared with 8.4% in propensity-matched controls [7]. Identifying those individuals at risk of cirrhosis before it develops would be of enormous utility.

Defining normal ranges for new biomarkers is essential if they are to be used to assess the presence, absence, or change of disease over time. There is an additional need for objective biomarkers to determine the efficacy of potential treatments, as there are currently no licensed pharmaceutical treatments for NASH. Furthermore, natural history studies of liver disease have been limited to small cohorts in the past because the only way of assessing liver disease across the spectrum from healthy to cirrhosis was with serial liver biopsy. In 1990, Powell et al. studied 42 NAFL patients with serial liver biopsies for a median of 4.5 years (range 1.5–20 years) and concluded that NASH is a cause of hepatic inflammation histologically resembling that of alcohol-induced liver disease but usually slowly progressive and of low-grade severity [29]. Ekstedt et al. studied 129 patients with biopsy-proven NAFL, whom they followed over 13 years, with repeat biopsies in 88 [30]. Disease progression in the liver occurred in 41% of the repeat biopsy patients, while 78% of the whole cohort developed diabetes, 19% died, and a further 4% had end-stage liver failure. However, there is no natural history study of liver-related outcomes in a low-risk population that can be used as a comparison. cT1 values have been shown to predict clinical outcomes in chronic liver disease patients [9]. The whole UK Biobank cohort will be censured at 3 yearly intervals to collect clinical outcomes. Our hypothesis is that those participants with high cT1 values are more likely to suffer clinical outcomes.

Our study also shows that age and gender have a negligible influence on cT1, with a maximum median value variation of 24.1 (658.3 vs. 692.4 ms). This suggests that, in practice, a correction of cT1 for age and gender may not be necessary. The small, but statistically significant, increase in cT1 in the youngest female group (age 40–49 years), which can be assumed to be largely pre-menopausal, is most likely explained by the effect of higher estrogen levels on liver water content, as previously described [31, 32].

While cT1 showed statistically significant correlations with both PDFF and T2*, these were weak (*r*^2^ = 0.45) and very weak (*r*^2^ = 0.004), respectively. Simulation studies have suggested a sequence-dependent positive effect of high fat on T1 [33], which may contribute to this weak correlation; however, steatosis often co-occurs with inflammation in liver diseases such as NASH [2] and viral hepatitis [34], based on histological assessment. Further studies are required to tease apart the relative contributions of these effects.

In this study, we describe the expected values of the cT1 in a healthy population, defined as a cohort at low risk for NAFL by the absence of a BMI ≥ 25 kg/m^2^ and hepatic steatosis. The value of this cohort is as a comparator for populations with greater likelihood of disease, for whom it can serve as a baseline.

## Limitations

The pilot phase of the UK Biobank imaging enhancement study has shown that high-throughput phenotyping is feasible. However, as MR methods develop (e.g., multispectral measurement of PDFF), further refinement of these techniques will need testing and implementing. The current ROI-based analysis from a single slice may have limitations in very heterogeneous disease distributions. In such cases, a histogram-based analysis may be more appropriate. A whole liver evaluation would allow better characterization of heterogeneity and reduce sampling errors; however, given the extremely high throughput of the study, there was insufficient time in the protocol to scan the whole liver. Given the UK Biobank subjects were drawn from a nominally healthy population, this is unlikely to significantly impact the conclusions of this study. The imaging data used in this study were acquired on a single scanner and therefore does not include any potential variability that may occur between different MRI scanners; however, computation of cT1 explicitly accounts for differences in field strength and T1 mapping implementation (LiverMultiScan version 2.0, Perspectum Diagnostics Ltd, UK). Approximately 4% of scans in this normal population were incomplete or of insufficient quantity, and this may increase when applied to an actual patient population.

The age range of the population is restricted to those of 40 years of age and above, so this population may not be representative of younger people. Also, the current definition of healthy solely in terms of BMI and hepatic steatosis does not account for other factors that can influence liver health such as alcohol and drug use, genetic predisposition, and infection. Information on ethnicity and other socio-economic factors is becoming available, so their influences should also be explored. As further UK Biobank data are released, more refined analyses will become possible.

## Conclusions

This article describes, for the first time, the reference range of cT1 values as defined in a large population at low risk for NAFL. The range presented here has the potential, where desirable, to serve as a benchmark of normality for future studies assessing NAFL with PDFF and cT1.

## Appendix

See Figs. 4, 5, 6, 7, and 8.
